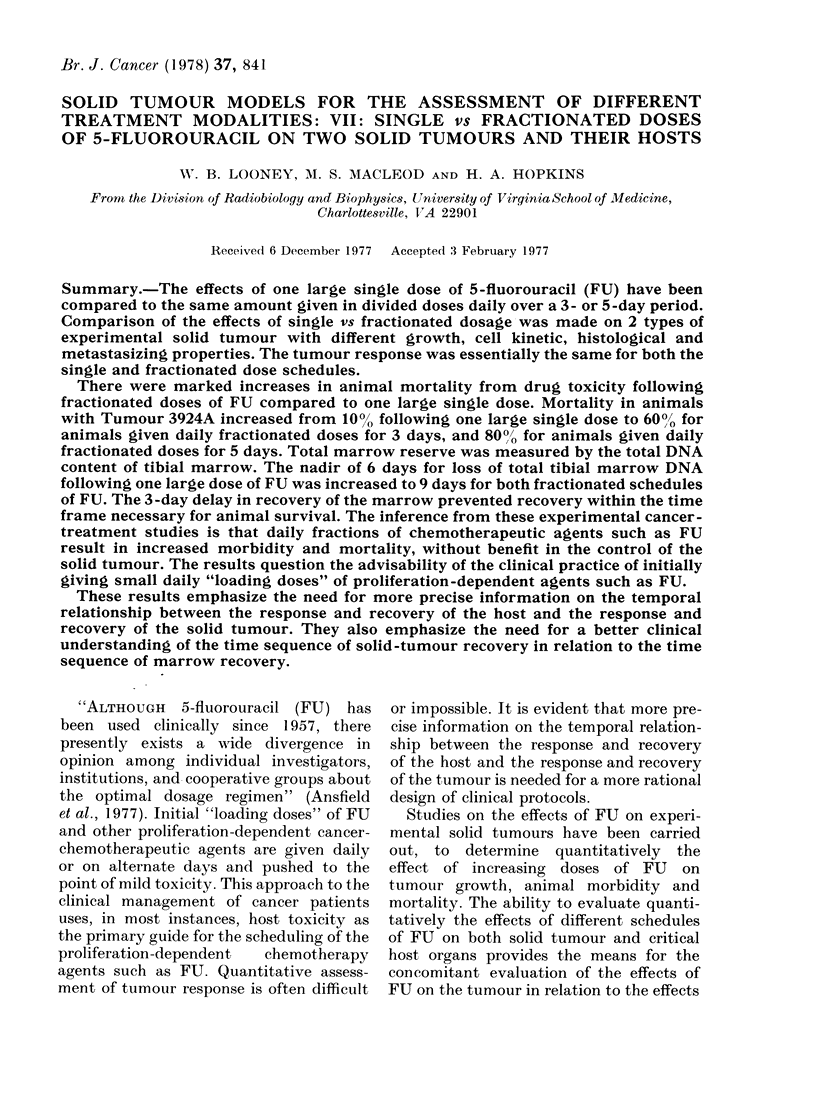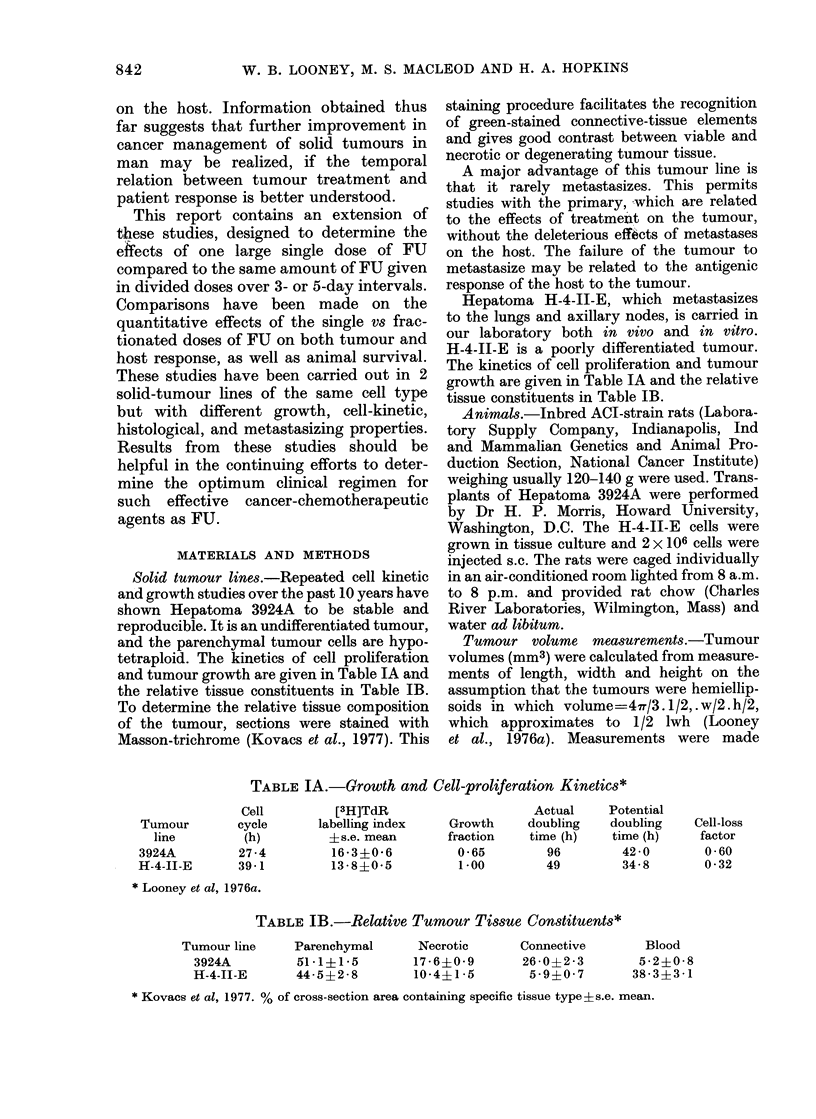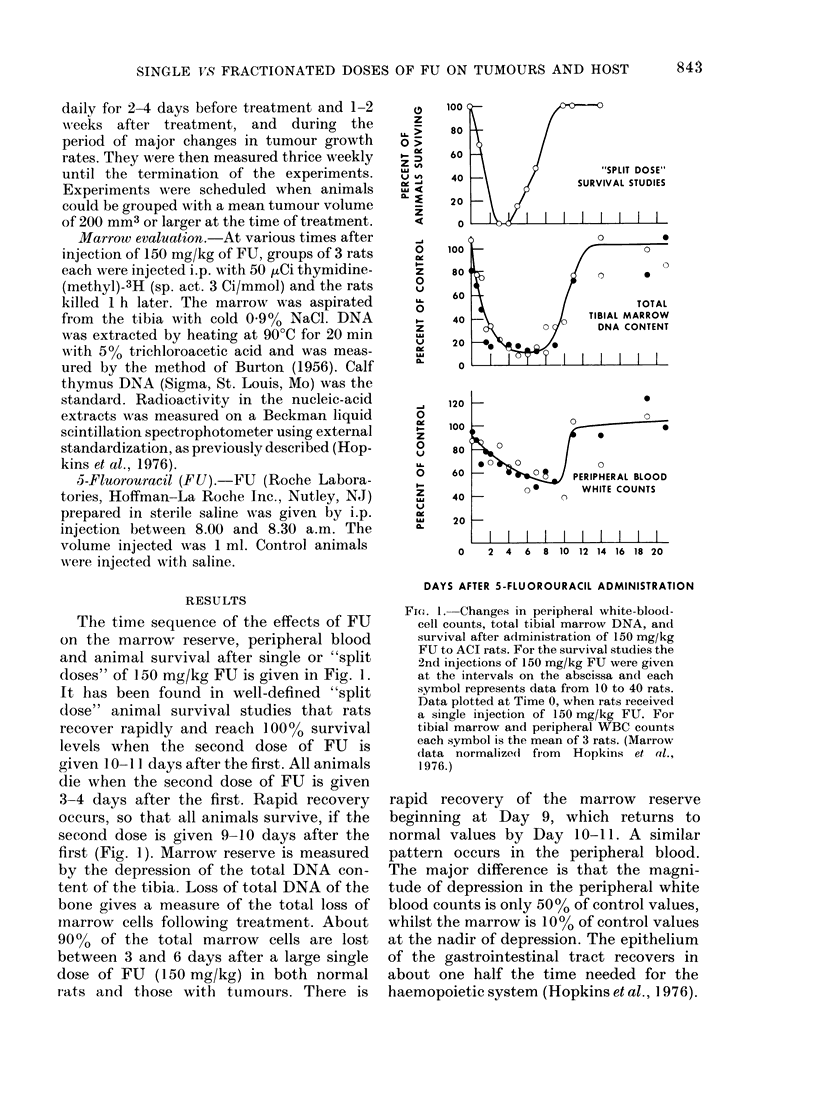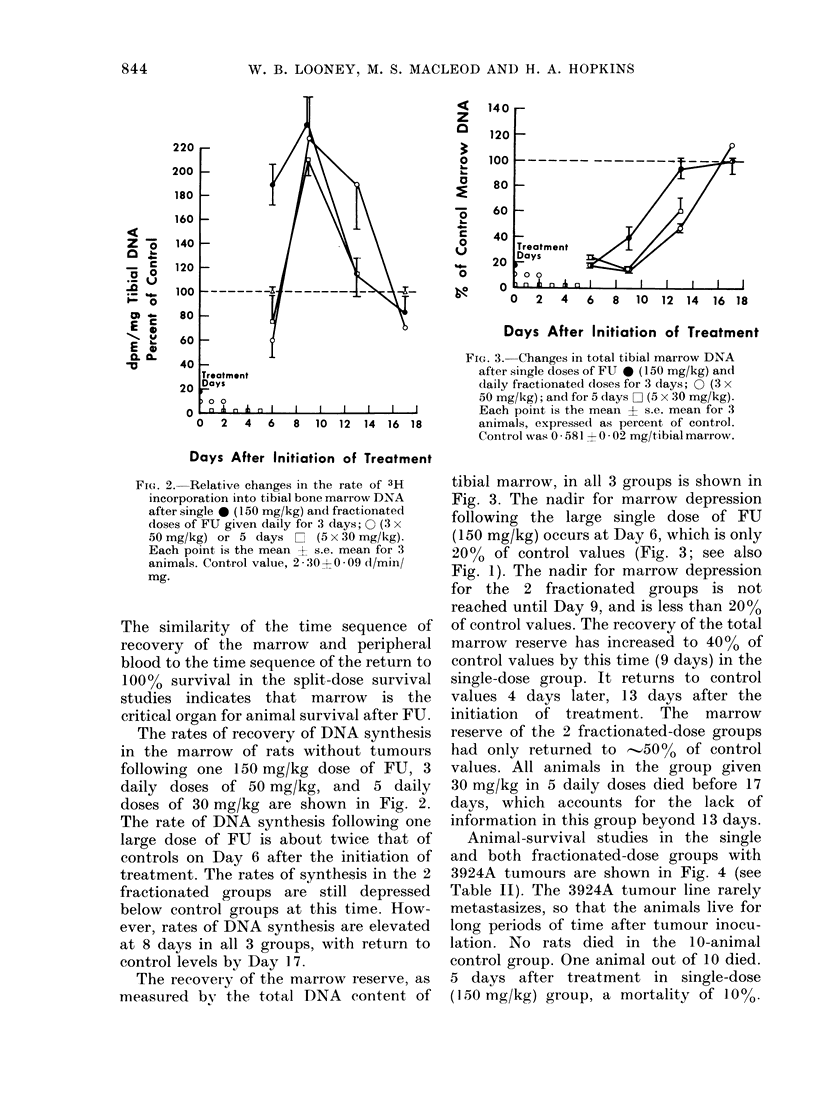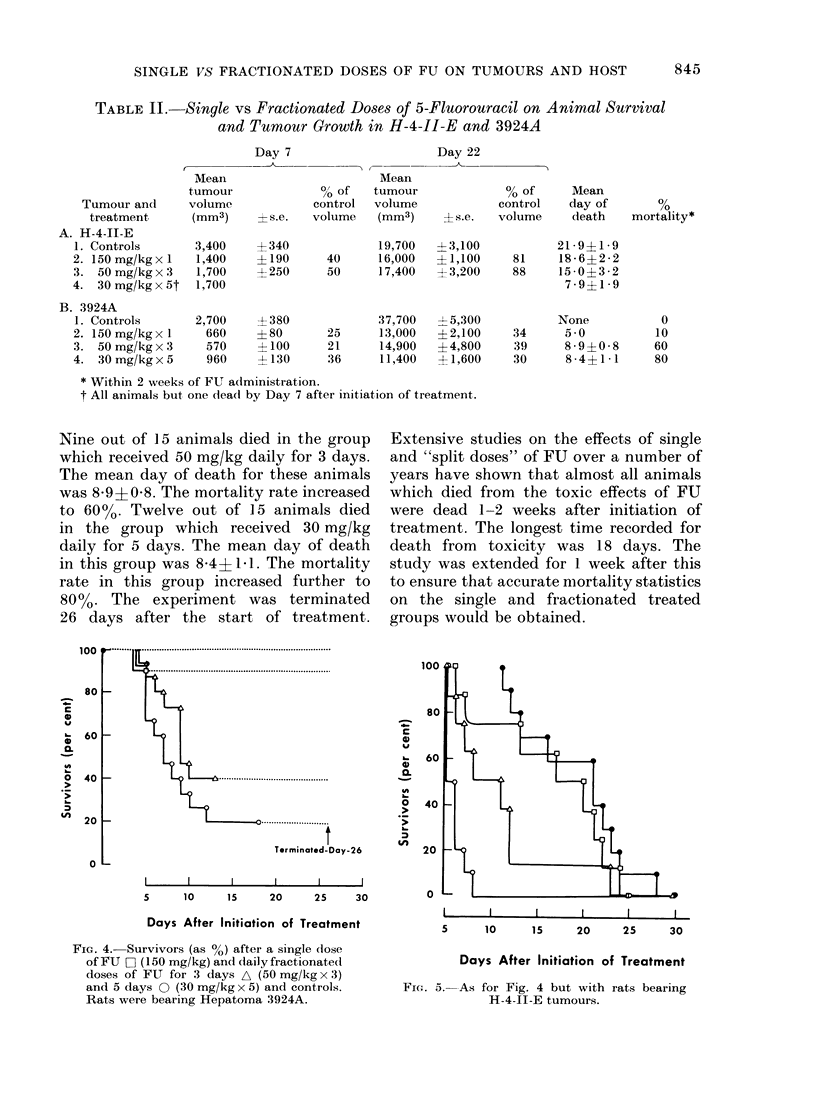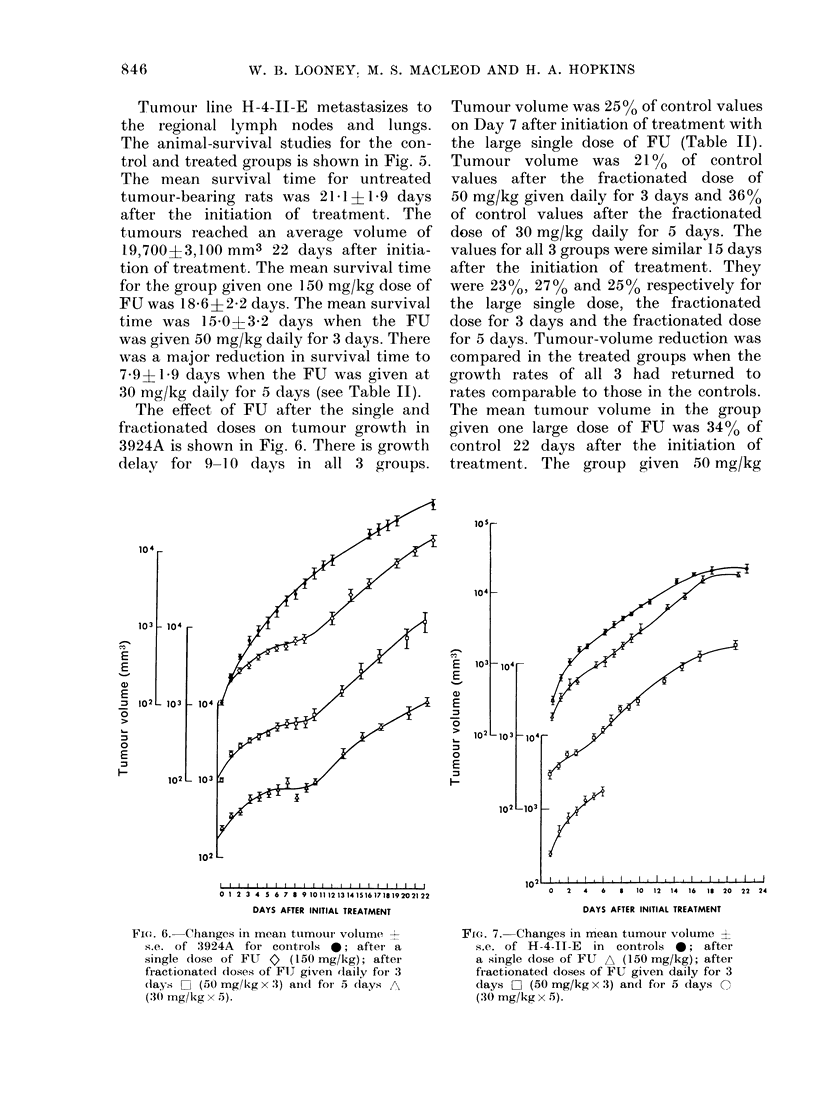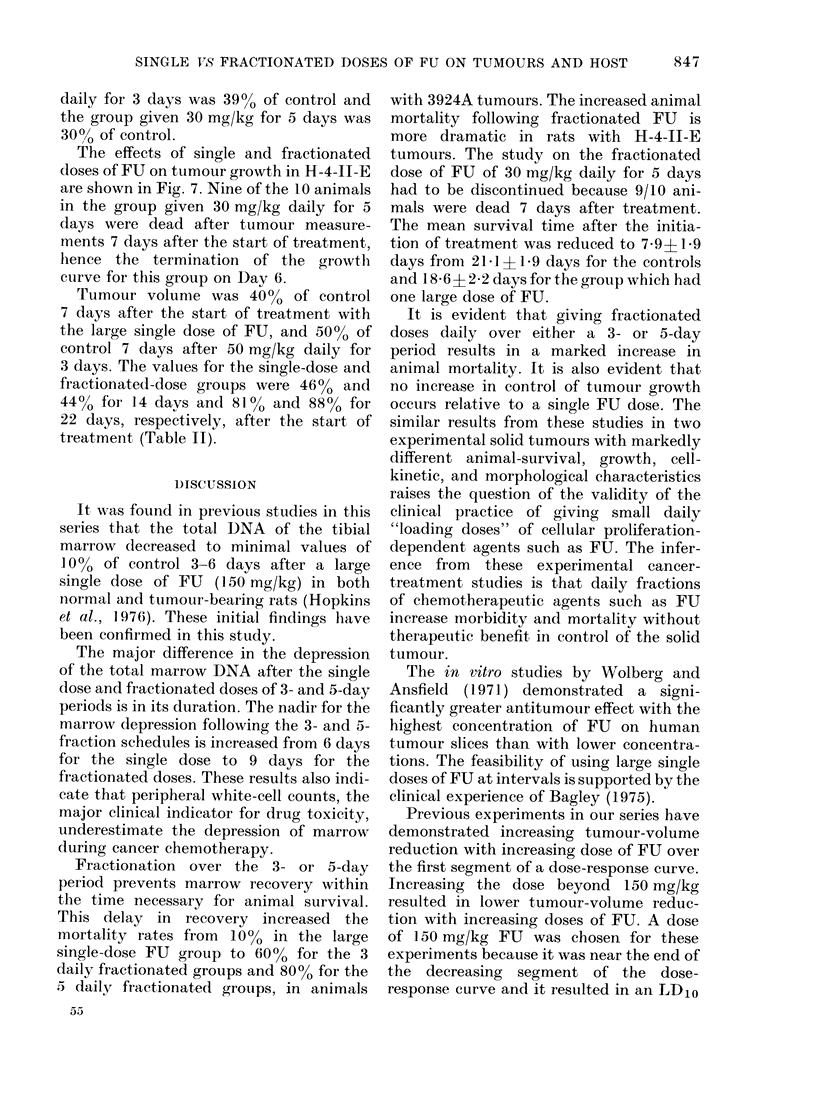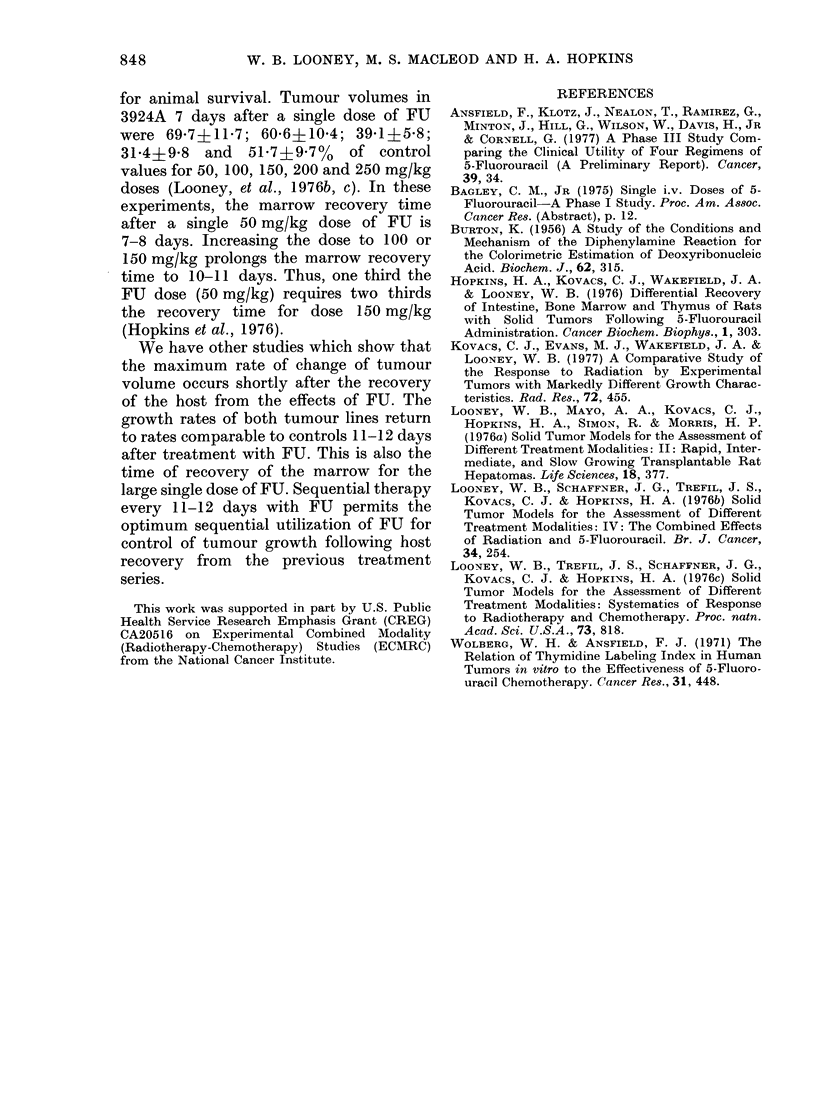# Solid Tumour Models for the Assessment of Different Treatment Modalities: VII: Single vs Fractionated Doses of 5-Fluorouracil on Two Solid Tumours and Their Hosts

**DOI:** 10.1038/bjc.1978.123

**Published:** 1978-05

**Authors:** W. B. Looney, M. S. MacLeod, H. A. Hopkins

## Abstract

The effects of one large single dose of 5-fluorouracil (FU) have been compared to the same amount given in divided doses daily over a 3- or 5-day period. Comparison of the effects of single *vs* fractionated dosage was made on 2 types of experimental solid tumour with different growth, cell kinetic, histological and metastasizing properties. The tumour response was essentially the same for both the single and fractionated dose schedules.

There were marked increases in animal mortality from drug toxicity following fractionated doses of FU compared to one large single dose. Mortality in animals with Tumour 3924A increased from 10% following one large single dose to 60% for animals given daily fractionated doses for 3 days, and 80% for animals given daily fractionated doses for 5 days. Total marrow reserve was measured by the total DNA content of tibial marrow. The nadir of 6 days for loss of total tibial marrow DNA following one large dose of FU was increased to 9 days for both fractionated schedules of FU. The 3-day delay in recovery of the marrow prevented recovery within the time frame necessary for animal survival. The inference from these experimental cancer-treatment studies is that daily fractions of chemotherapeutic agents such as FU result in increased morbidity and mortality, without benefit in the control of the solid tumour. The results question the advisability of the clinical practice of initially giving small daily “loading doses” of proliferation-dependent agents such as FU.

These results emphasize the need for more precise information on the temporal relationship between the response and recovery of the host and the response and recovery of the solid tumour. They also emphasize the need for a better clinical understanding of the time sequence of solid-tumour recovery in relation to the time sequence of marrow recovery.


*Br. J. Cancer* (1978) **37**, 841

### SOLID TUMOUR MODELS FOR THE ASSESSMENT OF DIFFERENT TREATMENT MODALITIES: VII: SINGLE *vs* FRACTIONATED DOSES OF 5-FLUOROURACIL ON TWO SOLID TUMOURS AND THEIR HOSTS

W. B. LOONEY, M. S. MACLEOD AND H. A. HOPKINS

*From the Division of Radiobiology and Biophysics, University of Virginia School of Medicine, Charlottesville, VA 22901*

Received 6 December 1977 Accepted 3 February 1977

**Summary.—The effects of one large single dose of 5-fluorouracil (FU) have been compared to the same amount given in divided doses daily over a 3- or 5-day period. Comparison of the effects of single *vs* fractionated dosage was made on 2 types of experimental solid tumour with different growth, cell kinetic, histological and metastasizing properties. The tumour response was essentially the same for both the single and fractionated dose schedules.**

**There were marked increases in animal mortality from drug toxicity following fractionated doses of FU compared to one large single dose. Mortality in animals with Tumour 3924A increased from 10% following one large single dose to 60% for animals given daily fractionated doses for 3 days, and 80% for animals given daily fractionated doses for 5 days. Total marrow reserve was measured by the total DNA content of tibial marrow. The nadir of 6 days for loss of total tibial marrow DNA following one large dose of FU was increased to 9 days for both fractionated schedules of FU. The 3-day delay in recovery of the marrow prevented recovery within the time frame necessary for animal survival. The inference from these experimental cancer-treatment studies is that daily fractions of chemotherapeutic agents such as FU result in increased morbidity and mortality, without benefit in the control of the solid tumour. The results question the advisability of the clinical practice of initially giving small daily "loading doses" of proliferation-dependent agents such as FU.**

**These results emphasize the need for more precise information on the temporal relationship between the response and recovery of the host and the response and recovery of the solid tumour. They also emphasize the need for a better clinical understanding of the time sequence of solid-tumour recovery in relation to the time sequence of marrow recovery.**

"ALTHOUGH 5-fluorouracil (FU) has been used clinically since 1957, there presently exists a wide divergence in opinion among individual investigators, institutions, and cooperative groups about the optimal dosage regimen" (Ansfield *et al.*, 1977). Initial "loading doses" of FU and other proliferation-dependent cancer-chemotherapeutic agents are given daily or on alternate days and pushed to the point of mild toxicity. This approach to the clinical management of cancer patients uses, in most instances, host toxicity as the primary guide for the scheduling of the proliferation-dependent chemotherapy agents such as FU. Quantitative assessment of tumour response is often difficult or impossible. It is evident that more precise information on the temporal relationship between the response and recovery of the host and the response and recovery of the tumour is needed for a more rational design of clinical protocols.

Studies on the effects of FU on experimental solid tumours have been carried out, to determine quantitatively the effect of increasing doses of FU on tumour growth, animal morbidity and mortality. The ability to evaluate quantitatively the effects of different schedules of FU on both solid tumour and critical host organs provides the means for the concomitant evaluation of the effects of FU on the tumour in relation to the effects on the host. Information obtained thus far suggests that further improvement in cancer management of solid tumours in man may be realized, if the temporal relation between tumour treatment and patient response is better understood.

This report contains an extension of these studies, designed to determine the effects of one large single dose of FU compared to the same amount of FU given in divided doses over 3- or 5-day intervals. Comparisons have been made on the quantitative effects of the single *vs* fractionated doses of FU on both tumour and host response, as well as animal survival. These studies have been carried out in 2 solid-tumour lines of the same cell type but with different growth, cell-kinetic, histological, and metastasizing properties. Results from these studies should be helpful in the continuing efforts to determine the optimum clinical regimen for such effective cancer-chemotherapeutic agents as FU.

### MATERIALS AND METHODS

*Solid tumour lines.*—Repeated cell kinetic and growth studies over the past 10 years have shown Hepatoma 3924A to be stable and reproducible. It is an undifferentiated tumour, and the parenchymal tumour cells are hypotetraploid. The kinetics of cell proliferation and tumour growth are given in Table IA and the relative tissue constituents in Table IB. To determine the relative tissue composition of the tumour, sections were stained with Masson-trichrome (Kovacs *et al.*, 1977). This staining procedure facilitates the recognition of green-stained connective-tissue elements and gives good contrast between viable and necrotic or degenerating tumour tissue.

A major advantage of this tumour line is that it rarely metastasizes. This permits studies with the primary, which are related to the effects of treatment on the tumour, without the deleterious effects of metastases on the host. The failure of the tumour to metastasize may be related to the antigenic response of the host to the tumour.

Hepatoma H-4-II-E, which metastasizes to the lungs and axillary nodes, is carried in our laboratory both *in vivo* and *in vitro*. H-4-II-E is a poorly differentiated tumour. The kinetics of cell proliferation and tumour growth are given in Table IA and the relative tissue constituents in Table IB.

*Animals.*—Inbred ACI-strain rats (Laboratory Supply Company, Indianapolis, Ind and Mammalian Genetics and Animal Production Section, National Cancer Institute) weighing usually 120–140 g were used. Transplants of Hepatoma 3924A were performed by Dr H. P. Morris, Howard University, Washington, D.C. The H-4-II-E cells were grown in tissue culture and $2 \times 10^6$ cells were injected s.c. The rats were caged individually in an air-conditioned room lighted from 8 a.m. to 8 p.m. and provided rat chow (Charles River Laboratories, Wilmington, Mass) and water *ad libitum*.

*Tumour volume measurements.*—Tumour volumes ($mm^3$) were calculated from measurements of length, width and height on the assumption that the tumours were hemiellipsoids in which volume$=4\pi/3.1/2,.w/2.h/2$, which approximates to 1/2 lwh (Looney *et al.*, 1976*a*). Measurements were made

TABLE IA.—*Growth and Cell-proliferation Kinetics**

| Tumour line | Cell cycle (h) | [$^3$H]TdR labelling index ±s.e. mean | Growth fraction | Actual doubling time (h) | Potential doubling time (h) | Cell-loss factor |
|---|---|---|---|---|---|---|
| 3924A | 27·4 | 16·3±0·6 | 0·65 | 96 | 42·0 | 0·60 |
| H-4-II-E | 39·1 | 13·8±0·5 | 1·00 | 49 | 34·8 | 0·32 |

* Looney *et al*, 1976*a*.

TABLE IB.—*Relative Tumour Tissue Constituents**

| Tumour line | Parenchymal | Necrotic | Connective | Blood |
|---|---|---|---|---|
| 3924A | 51·1±1·5 | 17·6±0·9 | 26·0±2·3 | 5·2±0·8 |
| H-4-II-E | 44·5±2·8 | 10·4±1·5 | 5·9±0·7 | 38·3±3·1 |

* Kovacs *et al*, 1977. % of cross-section area containing specific tissue type±s.e. mean.

daily for 2–4 days before treatment and 1–2 weeks after treatment, and during the period of major changes in tumour growth rates. They were then measured thrice weekly until the termination of the experiments. Experiments were scheduled when animals could be grouped with a mean tumour volume of 200 $mm^3$ or larger at the time of treatment.

*Marrow evaluation.*—At various times after injection of 150 mg/kg of FU, groups of 3 rats each were injected i.p. with 50 $\mu$Ci thymidine-(methyl)-$^3$H (sp. act. 3 Ci/mmol) and the rats killed 1 h later. The marrow was aspirated from the tibia with cold 0·9% NaCl. DNA was extracted by heating at 90°C for 20 min with 5% trichloroacetic acid and was measured by the method of Burton (1956). Calf thymus DNA (Sigma, St. Louis, Mo) was the standard. Radioactivity in the nucleic-acid extracts was measured on a Beckman liquid scintillation spectrophotometer using external standardization, as previously described (Hopkins *et al.*, 1976).

*5-Fluorouracil (FU).*—FU (Roche Laboratories, Hoffman-La Roche Inc., Nutley, NJ) prepared in sterile saline was given by i.p. injection between 8.00 and 8.30 a.m. The volume injected was 1 ml. Control animals were injected with saline.

FIG. 1.—Changes in peripheral white-blood-cell counts, total tibial marrow DNA, and survival after administration of 150 mg/kg FU to ACI rats. For the survival studies the 2nd injections of 150 mg/kg FU were given at the intervals on the abscissa and each symbol represents data from 10 to 40 rats. Data plotted at Time 0, when rats received a single injection of 150 mg/kg FU. For tibial marrow and peripheral WBC counts each symbol is the mean of 3 rats. (Marrow data normalized from Hopkins *et al.*, 1976.)

### RESULTS

The time sequence of the effects of FU on the marrow reserve, peripheral blood and animal survival after single or "split doses" of 150 mg/kg FU is given in Fig. 1. It has been found in well-defined "split dose" animal survival studies that rats recover rapidly and reach 100% survival levels when the second dose of FU is given 10–11 days after the first. All animals die when the second dose of FU is given 3–4 days after the first. Rapid recovery occurs, so that all animals survive, if the second dose is given 9–10 days after the first (Fig. 1). Marrow reserve is measured by the depression of the total DNA content of the tibia. Loss of total DNA of the bone gives a measure of the total loss of marrow cells following treatment. About 90% of the total marrow cells are lost between 3 and 6 days after a large single dose of FU (150 mg/kg) in both normal rats and those with tumours. There is rapid recovery of the marrow reserve beginning at Day 9, which returns to normal values by Day 10–11. A similar pattern occurs in the peripheral blood. The major difference is that the magnitude of depression in the peripheral white blood counts is only 50% of control values, whilst the marrow is 10% of control values at the nadir of depression. The epithelium of the gastrointestinal tract recovers in about one half the time needed for the haemopoietic system (Hopkins *et al.*, 1976).

FIG. 2.—Relative changes in the rate of $^{3}H$ incorporation into tibial bone marrow DNA after single ● (150 mg/kg) and fractionated doses of FU given daily for 3 days; ○ (3 × 50 mg/kg) or 5 days □ (5 × 30 mg/kg). Each point is the mean ± s.e. mean for 3 animals. Control value, 2·30±0·09 d/min/mg.

FIG. 3.—Changes in total tibial marrow DNA after single doses of FU ● (150 mg/kg) and daily fractionated doses for 3 days; ○ (3 × 50 mg/kg); and for 5 days □ (5 × 30 mg/kg). Each point is the mean ± s.e. mean for 3 animals, expressed as percent of control. Control was 0·581 ± 0·02 mg/tibial marrow.

The similarity of the time sequence of recovery of the marrow and peripheral blood to the time sequence of the return to 100% survival in the split-dose survival studies indicates that marrow is the critical organ for animal survival after FU.

The rates of recovery of DNA synthesis in the marrow of rats without tumours following one 150 mg/kg dose of FU, 3 daily doses of 50 mg/kg, and 5 daily doses of 30 mg/kg are shown in Fig. 2. The rate of DNA synthesis following one large dose of FU is about twice that of controls on Day 6 after the initiation of treatment. The rates of synthesis in the 2 fractionated groups are still depressed below control groups at this time. However, rates of DNA synthesis are elevated at 8 days in all 3 groups, with return to control levels by Day 17.

The recovery of the marrow reserve, as measured by the total DNA content of tibial marrow, in all 3 groups is shown in Fig. 3. The nadir for marrow depression following the large single dose of FU (150 mg/kg) occurs at Day 6, which is only 20% of control values (Fig. 3; see also Fig. 1). The nadir for marrow depression for the 2 fractionated groups is not reached until Day 9, and is less than 20% of control values. The recovery of the total marrow reserve has increased to 40% of control values by this time (9 days) in the single-dose group. It returns to control values 4 days later, 13 days after the initiation of treatment. The marrow reserve of the 2 fractionated-dose groups had only returned to ~50% of control values. All animals in the group given 30 mg/kg in 5 daily doses died before 17 days, which accounts for the lack of information in this group beyond 13 days.

Animal-survival studies in the single and both fractionated-dose groups with 3924A tumours are shown in Fig. 4 (see Table II). The 3924A tumour line rarely metastasizes, so that the animals live for long periods of time after tumour inoculation. No rats died in the 10-animal control group. One animal out of 10 died. 5 days after treatment in single-dose (150 mg/kg) group, a mortality of 10%.

TABLE II.—*Single* vs *Fractionated Doses of* 5-*Fluorouracil on Animal Survival and Tumour Growth in H*-4-*II*-*E and* 3924*A*

| | Day 7 | | | Day 22 | | | | |
|---|---|---|---|---|---|---|---|---|
| Tumour and treatment | Mean tumour volume ($mm^3$) | ±s.e. | % of control volume | Mean tumour volume ($mm^3$) | ±s.e. | % of control volume | Mean day of death | % mortality* |
| A. H-4-II-E | | | | | | | | |
| 1. Controls | 3,400 | ±340 | | 19,700 | ±3,100 | | 21·9±1·9 | |
| 2. 150 mg/kg × 1 | 1,400 | ±190 | 40 | 16,000 | ±1,100 | 81 | 18·6±2·2 | |
| 3. 50 mg/kg × 3 | 1,700 | ±250 | 50 | 17,400 | ±3,200 | 88 | 15·0±3·2 | |
| 4. 30 mg/kg × 5† | 1,700 | | | | | | 7·9±1·9 | |
| B. 3924A | | | | | | | | |
| 1. Controls | 2,700 | ±380 | | 37,700 | ±5,300 | | None | 0 |
| 2. 150 mg/kg × 1 | 660 | ±80 | 25 | 13,000 | ±2,100 | 34 | 5·0 | 10 |
| 3. 50 mg/kg × 3 | 570 | ±100 | 21 | 14,900 | ±4,800 | 39 | 8·9±0·8 | 60 |
| 4. 30 mg/kg × 5 | 960 | ±130 | 36 | 11,400 | ±1,600 | 30 | 8·4±1·1 | 80 |

* Within 2 weeks of FU administration.
† All animals but one dead by Day 7 after initiation of treatment.

Nine out of 15 animals died in the group which received 50 mg/kg daily for 3 days. The mean day of death for these animals was 8·9±0·8. The mortality rate increased to 60%. Twelve out of 15 animals died in the group which received 30 mg/kg daily for 5 days. The mean day of death in this group was 8·4±1·1. The mortality rate in this group increased further to 80%. The experiment was terminated 26 days after the start of treatment.

Extensive studies on the effects of single and "split doses" of FU over a number of years have shown that almost all animals which died from the toxic effects of FU were dead 1–2 weeks after initiation of treatment. The longest time recorded for death from toxicity was 18 days. The study was extended for 1 week after this to ensure that accurate mortality statistics on the single and fractionated treated groups would be obtained.

FIG. 4.—Survivors (as %) after a single dose of FU □ (150 mg/kg) and daily fractionated doses of FU for 3 days △ (50 mg/kg × 3) and 5 days ○ (30 mg/kg × 5) and controls. Rats were bearing Hepatoma 3924A.

FIG. 5.—As for Fig. 4 but with rats bearing H-4-II-E tumours.

Tumour line H-4-II-E metastasizes to the regional lymph nodes and lungs. The animal-survival studies for the control and treated groups is shown in Fig. 5. The mean survival time for untreated tumour-bearing rats was 21·1±1·9 days after the initiation of treatment. The tumours reached an average volume of 19,700±3,100 mm$^3$ 22 days after initiation of treatment. The mean survival time for the group given one 150 mg/kg dose of FU was 18·6±2·2 days. The mean survival time was 15·0±3·2 days when the FU was given 50 mg/kg daily for 3 days. There was a major reduction in survival time to 7·9±1·9 days when the FU was given at 30 mg/kg daily for 5 days (see Table II).

The effect of FU after the single and fractionated doses on tumour growth in 3924A is shown in Fig. 6. There is growth delay for 9–10 days in all 3 groups. Tumour volume was 25% of control values on Day 7 after initiation of treatment with the large single dose of FU (Table II). Tumour volume was 21% of control values after the fractionated dose of 50 mg/kg given daily for 3 days and 36% of control values after the fractionated dose of 30 mg/kg daily for 5 days. The values for all 3 groups were similar 15 days after the initiation of treatment. They were 23%, 27% and 25% respectively for the large single dose, the fractionated dose for 3 days and the fractionated dose for 5 days. Tumour-volume reduction was compared in the treated groups when the growth rates of all 3 had returned to rates comparable to those in the controls. The mean tumour volume in the group given one large dose of FU was 34% of control 22 days after the initiation of treatment. The group given 50 mg/kg

FIG. 6.—Changes in mean tumour volume ± s.e. of 3924A for controls ●; after a single dose of FU ◇ (150 mg/kg); after fractionated doses of FU given daily for 3 days □ (50 mg/kg × 3) and for 5 days △ (30 mg/kg × 5).

FIG. 7.—Changes in mean tumour volume ± s.e. of H-4-II-E in controls ●; after a single dose of FU △ (150 mg/kg); after fractionated doses of FU given daily for 3 days □ (50 mg/kg × 3) and for 5 days ○ (30 mg/kg × 5).

daily for 3 days was 39% of control and the group given 30 mg/kg for 5 days was 30% of control.

The effects of single and fractionated doses of FU on tumour growth in H-4-II-E are shown in Fig. 7. Nine of the 10 animals in the group given 30 mg/kg daily for 5 days were dead after tumour measurements 7 days after the start of treatment, hence the termination of the growth curve for this group on Day 6.

Tumour volume was 40% of control 7 days after the start of treatment with the large single dose of FU, and 50% of control 7 days after 50 mg/kg daily for 3 days. The values for the single-dose and fractionated-dose groups were 46% and 44% for 14 days and 81% and 88% for 22 days, respectively, after the start of treatment (Table II).

### DISCUSSION

It was found in previous studies in this series that the total DNA of the tibial marrow decreased to minimal values of 10% of control 3–6 days after a large single dose of FU (150 mg/kg) in both normal and tumour-bearing rats (Hopkins *et al.*, 1976). These initial findings have been confirmed in this study.

The major difference in the depression of the total marrow DNA after the single dose and fractionated doses of 3- and 5-day periods is in its duration. The nadir for the marrow depression following the 3- and 5-fraction schedules is increased from 6 days for the single dose to 9 days for the fractionated doses. These results also indicate that peripheral white-cell counts, the major clinical indicator for drug toxicity, underestimate the depression of marrow during cancer chemotherapy.

Fractionation over the 3- or 5-day period prevents marrow recovery within the time necessary for animal survival. This delay in recovery increased the mortality rates from 10% in the large single-dose FU group to 60% for the 3 daily fractionated groups and 80% for the 5 daily fractionated groups, in animals with 3924A tumours. The increased animal mortality following fractionated FU is more dramatic in rats with H-4-II-E tumours. The study on the fractionated dose of FU of 30 mg/kg daily for 5 days had to be discontinued because 9/10 animals were dead 7 days after treatment. The mean survival time after the initiation of treatment was reduced to $7{\cdot}9 \pm 1{\cdot}9$ days from $21{\cdot}1 \pm 1{\cdot}9$ days for the controls and $18{\cdot}6 \pm 2{\cdot}2$ days for the group which had one large dose of FU.

It is evident that giving fractionated doses daily over either a 3- or 5-day period results in a marked increase in animal mortality. It is also evident that no increase in control of tumour growth occurs relative to a single FU dose. The similar results from these studies in two experimental solid tumours with markedly different animal-survival, growth, cell-kinetic, and morphological characteristics raises the question of the validity of the clinical practice of giving small daily "loading doses" of cellular proliferation-dependent agents such as FU. The inference from these experimental cancer-treatment studies is that daily fractions of chemotherapeutic agents such as FU increase morbidity and mortality without therapeutic benefit in control of the solid tumour.

The *in vitro* studies by Wolberg and Ansfield (1971) demonstrated a significantly greater antitumour effect with the highest concentration of FU on human tumour slices than with lower concentrations. The feasibility of using large single doses of FU at intervals is supported by the clinical experience of Bagley (1975).

Previous experiments in our series have demonstrated increasing tumour-volume reduction with increasing dose of FU over the first segment of a dose-response curve. Increasing the dose beyond 150 mg/kg resulted in lower tumour-volume reduction with increasing doses of FU. A dose of 150 mg/kg FU was chosen for these experiments because it was near the end of the decreasing segment of the dose-response curve and it resulted in an $LD_{10}$

for animal survival. Tumour volumes in 3924A 7 days after a single dose of FU were 69·7±11·7; 60·6±10·4; 39·1±5·8; 31·4±9·8 and 51·7±9·7% of control values for 50, 100, 150, 200 and 250 mg/kg doses (Looney, *et al.*, 1976*b*, *c*). In these experiments, the marrow recovery time after a single 50 mg/kg dose of FU is 7–8 days. Increasing the dose to 100 or 150 mg/kg prolongs the marrow recovery time to 10–11 days. Thus, one third the FU dose (50 mg/kg) requires two thirds the recovery time for dose 150 mg/kg (Hopkins *et al.*, 1976).

We have other studies which show that the maximum rate of change of tumour volume occurs shortly after the recovery of the host from the effects of FU. The growth rates of both tumour lines return to rates comparable to controls 11–12 days after treatment with FU. This is also the time of recovery of the marrow for the large single dose of FU. Sequential therapy every 11–12 days with FU permits the optimum sequential utilization of FU for control of tumour growth following host recovery from the previous treatment series.

This work was supported in part by U.S. Public Health Service Research Emphasis Grant (CREG) CA20516 on Experimental Combined Modality (Radiotherapy-Chemotherapy) Studies (ECMRC) from the National Cancer Institute.

### REFERENCES

ANSFIELD, F., KLOTZ, J., NEALON, T., RAMIREZ, G., MINTON, J., HILL, G., WILSON, W., DAVIS, H., JR & CORNELL, G. (1977) A Phase III Study Comparing the Clinical Utility of Four Regimens of 5-Fluorouracil (A Preliminary Report). *Cancer*, **39**, 34.

BAGLEY, C. M., JR (1975) Single i.v. Doses of 5-Fluorouracil—A Phase I Study. *Proc. Am. Assoc. Cancer Res.* (Abstract), p. 12.

BURTON, K. (1956) A Study of the Conditions and Mechanism of the Diphenylamine Reaction for the Colorimetric Estimation of Deoxyribonucleic Acid. *Biochem. J.*, **62**, 315.

HOPKINS, H. A., KOVACS, C. J., WAKEFIELD, J. A. & LOONEY, W. B. (1976) Differential Recovery of Intestine, Bone Marrow and Thymus of Rats with Solid Tumors Following 5-Fluorouracil Administration. *Cancer Biochem. Biophys.*, **1**, 303.

KOVACS, C. J., EVANS, M. J., WAKEFIELD, J. A. & LOONEY, W. B. (1977) A Comparative Study of the Response to Radiation by Experimental Tumors with Markedly Different Growth Characteristics. *Rad. Res.*, **72**, 455.

LOONEY, W. B., MAYO, A. A., KOVACS, C. J., HOPKINS, H. A., SIMON, R. & MORRIS, H. P. (1976*a*) Solid Tumor Models for the Assessment of Different Treatment Modalities: II: Rapid, Intermediate, and Slow Growing Transplantable Rat Hepatomas. *Life Sciences*, **18**, 377.

LOONEY, W. B., SCHAFFNER, J. G., TREFIL, J. S., KOVACS, C. J. & HOPKINS, H. A. (1976*b*) Solid Tumor Models for the Assessment of Different Treatment Modalities: IV: The Combined Effects of Radiation and 5-Fluorouracil. *Br. J. Cancer*, **34**, 254.

LOONEY, W. B., TREFIL, J. S., SCHAFFNER, J. G., KOVACS, C. J. & HOPKINS, H. A. (1976*c*) Solid Tumor Models for the Assessment of Different Treatment Modalities: Systematics of Response to Radiotherapy and Chemotherapy. *Proc. natn. Acad. Sci. U.S.A.*, **73**, 818.

WOLBERG, W. H. & ANSFIELD, F. J. (1971) The Relation of Thymidine Labeling Index in Human Tumors *in vitro* to the Effectiveness of 5-Fluorouracil Chemotherapy. *Cancer Res.*, **31**, 448.